# Response to comment on ‘Hydrogen bonds in crystalline d-alanine: diffraction and spectroscopic evidence for differences between enantiomers’

**DOI:** 10.1107/S2052252518010321

**Published:** 2018-07-27

**Authors:** Ezequiel A. Belo, Jose E. M. Pereira, Paulo T. C. Freire, Dimitri N. Argyriou, Juergen Eckert, Heloisa N. Bordallo

**Affiliations:** a Faculdade de Física, Universidade Federal do Pará, Belém, Pará, Brazil; b Departamento de Física, Universidade Federal do Ceará, Fortaleza, Ceará, Brazil; c Niels Bohr Institute, University of Copenhagen, Universitetsparken 5, Copenhagen, 2100, Denmark; d European Spallation Source, 176, SE-221 00 Lund, Sweden; e Department of Chemistry, University of South Florida, 4202 East Fowler Ave, Tampa, FL 33620, USA; f Theoretical Division, Los Alamos National Laboratory, Los Alamos, NM 87545, USA

**Keywords:** chirality, structure analysis, configurational change, phase transitions, intermolecular interactions, amino acids

## Abstract

A response is given to comments by Bürgi & Macchi [*IUCrJ* (2018), **5**, 654–657] about Belo *et al.* [*IUCrJ* (2018), **5**, 6–12.].

In the preceding comment on our paper Bürgi & Macchi (2018 ▸) stated ‘The recent paper by Belo, Pereira, Freire, Argyriou, Eckert & Bordallo [(2018 ▸), *IUCrJ*, **5**, 6–12] reports observations that may lead one to think of very strong and visible consequences of the parity-violation energy difference between enantiomers of a molecule, namely alanine’ and ‘Therefore, the conclusions drawn by Belo *et al.* (2018 ▸) are deemed inappropriate as the data presented do not contain sufficient information to reach such a conclusion’. In response to this comment we would like to stress the point that we did not in fact draw any conclusions at all in our paper concerning the parity-violating energy difference (PVED) hypothesis of Salam [Salam (1992 ▸), see also Laerdahl *et al.* (2000 ▸) and Berger & Quack (2000 ▸) for discussion] and therefore find it difficult to see how they could therefore be ‘deemed inappropriate’.

Belo *et al.* (2018 ▸) reports a careful parametric (temperature-dependence) study of d-alanine by polarized single-crystal Raman spectroscopy and neutron powder diffraction and makes comparisons with results on both l- and d-alanine drawn from the literature. At temperatures where the structural information from the reported neutron powder diffraction measurements can be compared with previous single-crystal X-ray diffraction in l-alanine (Lehmann *et al.*, 1972 ▸; Destro *et al.*, 2008 ▸) and single-crystal neutron diffraction in l- and d-alanine (Wilson *et al.*, 2005 ▸), there is good agreement when the difference between hydrogenated and deuterated samples is taken into account. The results reported by Belo *et al.* (2018 ▸), however, provide a continuous picture of the temperature evolution of the bonds in d-alanine from 280 K down to 4 K, which shows that while the average structure is kept the same (no changes in space group) in d-alanine, as opposed to l-alanine, local symmetry changes are seen at lower temperatures. Furthermore, below 250 K, both l-alanine and d-alanine appear to undergo micro-conformational transitions resulting from a subtle rearrangement of the hydrogen-bond network. This temperature corresponds with that where bulk measurements (Barthès *et al.*, 2002 ▸, 2003 ▸; Wang *et al.*, 2000 ▸, 2002 ▸; Sullivan *et al.*, 2003 ▸) have observed anomalies that were indicative of a phase transition. Although it should be noted that Sullivan *et al.* (2003 ▸) were able to reduce this anomaly by re-growing their sample, it has been observed in a number of different samples prepared by different groups and should therefore be considered to be a real effect of as yet undetermined nature.

We note that the results of Belo *et al.* (2018 ▸) do not provide, or claim to provide, evidence for, or against, the Salam hypothesis, which predicted that quantum mechanical cooperative and condensation phenomena may give rise to a second-order phase transition below a critical temperature linking the transformation of d-amino acids to l-amino acids. An order of magnitude estimate by Salam (1992 ▸) indicated a transition temperature of ~250 K. The work of Belo *et al.* (2018 ▸) does not support the idea of the d-alanine (d-ala) → l-alanine (l-ala) transformation, but instead provides a microscopic picture of the alanine solids consistent with the other experimental measurements. The properties of l- and d-alanine, and the l- and d-amino acids in general, are a fascinating, and important, area of study for our understanding of nature, irrespective of whether they are related, or not, to the weak nuclear force and parity violation.
